# The impact of foot arch height on quality of life in 6-12 year olds

**Published:** 2014-12-30

**Authors:** Daniel López López, Mª de los Ángeles Bouza Prego, Ana Requeijo Constenla, Jesús Luis Saleta Canosa, Adolfo Bautista Casasnovas, Francisco Alonso Tajes

**Affiliations:** 1 Unidade Investigación Saúde e Podoloxía. Departamento de Ciencias da Saúde. Facultade de Enfermaría e Podoloxía. Universidade da Coruña, España; 2 Departamento de Pediatría. Facultad de Medicina y Odontología. Universidad de Santiago de Compostela. España

**Keywords:** Child care, foot deformities, foot disease, flat foot, Child welfare, school health services

## Abstract

**Objective:**

To determine whether arch height has an effect on the health-related quality of life of schoolchildren.

**Methods:**

One hundred and thirteen schoolchildren attended an out-patient centre where self-reported data were recorded, their feet were classified into one of three groups according to their arch index (high, normal or low) and the scores obtained from the Foot Health Status Questionnaire (FHSQ - Spanish version) were compared.

**Results:**

The groups with high, low and normal arch recorded lower scores in Section One for the general foot health and footwear domains and higher scores in foot pain and foot function. In Section Two they obtained lower scores in general health and higher scores in physical activity, social capacity and vigour.

**Conclusions:**

Comparison of the scores obtained reveals that arch height has a negative impact on quality of life. Given the limited extent of available evidence in respect of the aetiology and treatment of foot diseases and deformities, these findings reveal the need to implement programmes to promote foot health and carry out further research into this commonly occurring disabling condition.

## Abstract (es)

**Objetivo:**

Conocer si la altura del arco del pie tiene impacto en la calidad de vida relacionada con la salud en la etapa escolar.

**Métodos:**

Ciento trece escolares acudieron a un centro ambulatorio donde se registraron datos autoinformados, se clasificaron los pies en función de los valores del índice del arco en tres grupos (aumentado, disminuido y normal) y se compararon las puntuaciones obtenidas según el Foot Health Status Questionnaire (FHSQ) versión española.

**Resultados:**

Los grupos con arco del pie aumentado, disminuido y normal muestran puntuaciones más bajas en la primera sección en las dimensiones salud del pie y calzado y puntuaciones altas en dolor y función del pie. En la segunda sección obtuvieron menos puntuación en la dimensión salud general y puntuaciones altas en función física, función social y vitalidad.

**Conclusiones:**

La comparación de las puntuaciones obtenidas muestra que la altura del arco tiene un impacto negativo en la calidad de vida. Dado que la evidencia actual sobre la etiología y el tratamiento de las enfermedades y deformidades es limitada, estos resultados ponen de manifiesto la necesidad de implementar programas para promover la salud de los pies y seguir investigando en esta condición común e incapacitante.

## Introduction

Foot problems appear in approximately 70% to 80% of adults and 30% of children 1. The most commonly occurring problems concern deformities of the medial longitudinal arch, either because it is excessively high (a condition known as *pes cavus *or cavus foot) or excessively low (*pes planus *or flat foot) 2 ^-^ 4 and have a significant impact on foot function and the development of musculoskeletal pathologies 5 ^,^ 6; they are also believed to have a negative effect on quality of life 7.

Studies have revealed that 60% of the school-age population have normal arches, 20% have a high-arched foot and the remaining 20% a low medial arch 8. Hence the interest for health care in general, and podiatry in particular, in studying, detecting, assessing and treating foot alterations and deformities, especially those of the medial arch, with a view to preventing future injuries, improving children's quality of life and avoiding the appearance of problems in later life. If left untreated during childhood, foot deformities can lead to scoliosis, postural problems, slower walking speed, uneven plantar pressure distribution, difficulty in carrying out daily activities, an increased risk of falling and the appearance of neurological diseases 9 ^,^ 10, all of which affect quality of life, personal autonomy and well-being 11.

One of the most commonly used criteria among clinicians when classifying, studying and evaluating human biomechanics and foot characteristics is based on quantification of the longitudinal medial arch by the arch index (AI) method (IA) 12.

This indirect technique is one of the most widely described and cited in the literature. Its importance derives from its reliability in measuring foot characteristics 13, since in comparison with other measurements it produces fewer cases of non-classification 14. Furthermore, it quantifies the structure of the medial longitudinal arch, a feature of great significance since the latter is a predisposing factor for the appearance of injuries 15 ^,^ 16. Measuring the arch index therefore makes it possible to plan treatments to improve or maintain the medial arch and prevent the appearance of foot injuries 17 ^,^ 18.

With this background in mind, and taking into account the fact that the target population of this study are of school age, the detection of postural foot alterations and deformities, together with that of basic diseases, are factors that need to be considered when planning preventive treatments and activities with the aim of improving schoolchildren's quality of life and wellbeing. The goal of the study is therefore to determine whether foot arch height affects health-related quality of life in children of school age, since at present little is known about the factors that affect the development of the medial arch, which is in turn a predisposing factor for the appearance of injuries in later life that could be prevented by implementing programmes to improve the general condition of children's feet.

## Materials and Methods 

### Participants

A total of 113 primary schoolchildren of similar socio-economic level took part in a descriptive observational study carried out at an out-patient centre in the province of A Coruña (Spain) during the period January - December 2013.

Subjects were selected to participate in the study by means of the non-probability consecutive sampling technique. The ages of the children included in the study ranged from 6 to 12, and participants were excluded for the following reasons: severe prior trauma modifying foot morphology, previous foot surgery and alterations or deformities other than arch height, refusal to sign the informed consent document and the inability to understand the instructions given in the study and complete the required information. The research was approved by the Research and Ethics Committee of the Universidade da Coruña (Spain), file number CE 15/2013. All the parents and/or legal guardians gave their informed consent before the minors concerned were included in the study, with the ethical standards in human experimentation contained in the WMA Declaration of Helsinki, the Council of Europe Convention on Human Rights and Biomedicine, the UNESCO Universal Declaration on the Human Genome and Human Rights and those of the relevant national bodies and institutions being observed at all times. 

### Procedure

Measurements were made by a single clinic, which first measured the children's height and weight, calculated the body mass index (BMI) and correlated it with gender and the percentile in which the BMI fell. A child was considered to be overweight or obese when his or her BMI fell in the ≥85 percentile 19. Participants then completed the FHSQ questionnaire 18. This health-related quality of life self-reported instrument is specific to the foot and comprises three main sections. Section One assesses foot pain, foot function, footwear and general foot health. Section Two looks at general health, physical activity, social capacity and vigour, whilst Section Three focuses on socio-demographic data such as age, gender and injury history. 

**Figure 1.**
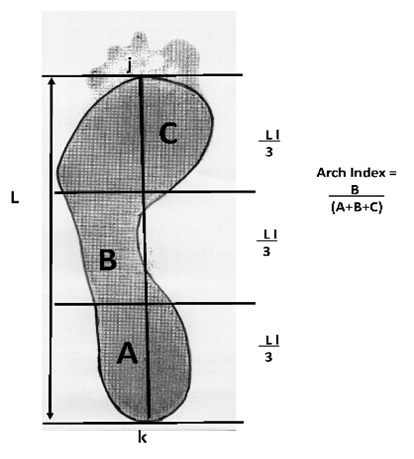
Outline of the footprint, excluding the toes, is traced with a scanner using AutoCad^®^ 2013. It is then divided into into three equal sections by the parallel lines perpendicular to j and k. The arch index (AI) is calculated as the relation between the midfoot area B and the area of the whole foot excluding the toes (A + B + C).

Static footprints in bipodal position were then taken by the photopodogram method 20 ^,^ 21. This technique consists of moistening the sole of the foot with photographic developer liquid and standing in bipodal position on black and white photographic paper on a flat horizontal surface for approximately 60 s 22. The footprints thus obtained were scanned and analysed with AutoCAD 14 software to measure the surface area of the foot in contact with the photographic paper 23 ^,^ 24. The program was then used to obtain the AI by measuring the area of the forefoot, midfoot and hindfoot, excluding the toes 12. The following values determine the type of foot: an IA of <0.21 is indicative of a low arch, a normal arch would have an IA of 0.21-0.26 and an IA of >0.26 is considered to indicate a high arch (Fig. 1).

The IBM software package SPSS^®^ Statistics 19 for Windows was used to analyse the data and provide a description of the variables included in the study. Qualitative variables are shown as absolute values and percentages, whilst in the case of quantitative variables the mean and standard deviation (SD) are given. Comparison of mean values was obtained by an analysis of variance (ANOVA) for independent samples. Scores for foot health related quality of life were obtained from the Foot Health Status Questionnaire Data Analysis^®^ Software Version 1.03. 

## Results 

A total of 113 schoolchildren completed all the stages of the research. Age, gender, height, weight and group percentiles are shown in Table 1. No significant differences were found between groups.

**Table 1.**
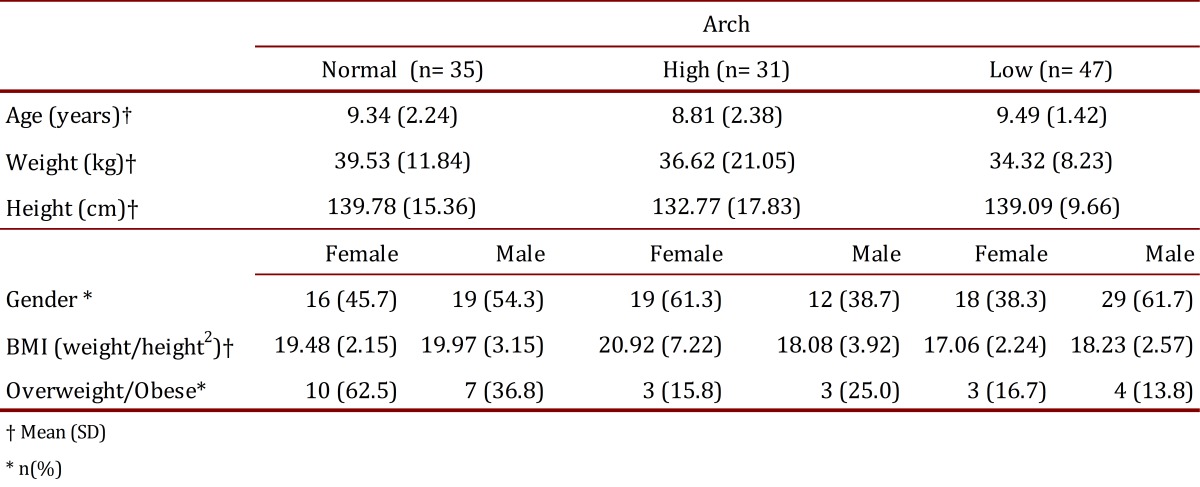
Socio-demographic and clinical characteristics of the sample population.

The sample size is clinically relevant, the results showing a difference of at least 21 points in the FHSQ between the groups studied, and given that the standard deviation for the arch scale in children is in the region of 29 points 25, in the case of a bilateral test with an alpha risk of 5% and a statistical power of 80%, a minimum of 31 subjects are needed in each group (n= 93).

Table 2 shows the relation between foot category and the FHSQ scores for the domains studied. As can be observed, there is no statistically significant relation between foot arch type and the various quality of life related domains of foot health, although low scores were reported in all groups for foot health, footwear and overall health in comparison with the scores recorded for the other domains.

**Table 2.**
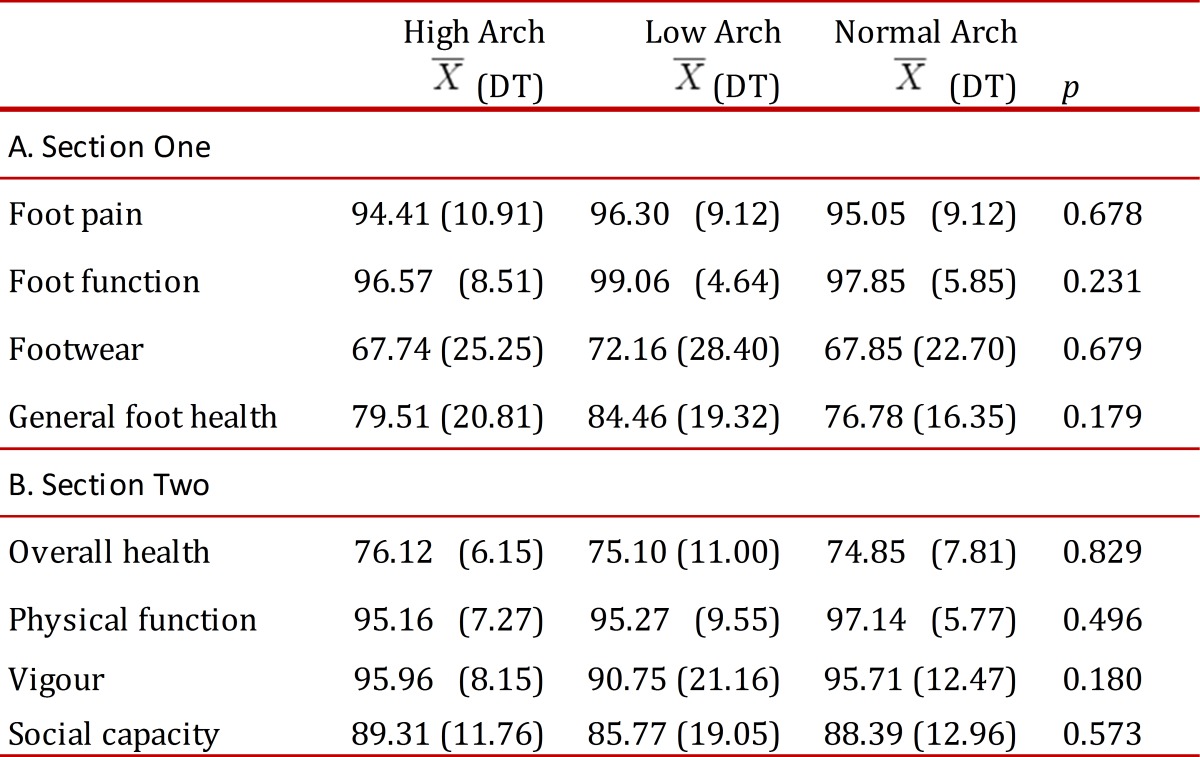
The relation between foot type (high, normal and low arch) and FHSQ scores.

The results of a comparison between FHSQ scores of the three groups within the sample population are shown in Table 2A and 2B. Section One of the FHSQ (Table 2A) evaluates four specific foot domains, namely pain, function, health and footwear. Mean scores ± SD were significantly high in the assessment of pain and function, and lower in foot health and footwear for all groups. Section Two (Table 2B) gives an assessment of four domains of general wellbeing: overall health, physical function, social capacity and vigour. In this case mean scores ± SD were significantly lower in the overall health domain when compared to those for other domains for all three groups: high arch, normal arch and low arch.

## Discussion

The main aim of this study was to determine whether foot arch height affects health-related quality of life, by comparing the self-reported FHSQ scores of a sample population of schoolchildren in three foot categories: high arch, normal arch and low arch. Before entering into a detailed discussion of the results, it is worth considering to what extent these three groups are representative in the school-age population as a whole and whether the similar representation shown by children of both genders is consistent with earlier reports in the literature that have related foot arch height with plantar pressure 16, strength with foot arch height 26 and three-dimensional measuring of the foot arch 27, concluding that variations in arch height may lead to foot problems. 

By definition, the participants with high and low arches were categorised according to the measurements obtained in the AI test, as were those with normal arches, in agreement with studies that have evaluated the foot arch to show standard growth of the foot arch and contributed to the diagnosis and treatment of foot alterations and deformities 28 ^,^ 29.

Comparison of the FHSQ scores (in terms of mean score ± SD) revealed similar results for the three groups of participants: high arch, low arch and normal arch. Foot health related quality of life, as the scores for Section One indicate, is comparatively low in the footwear and general foot health domains, proving to be independent from the effect of the BMI percentile of the respondents. These findings indicate that schoolchildren experience more foot pain, greater restrictions in terms of footwear and consider that their feet are in a worse state of health, with no differences as regards foot arch height 25. This may be related to a higher number of foot injuries amongst schoolchildren who engage in sports activities and register hypermobility 30 ^,^ 31, highlighting the need for regular foot care and monitoring.

Comparison of the impact of these scores for Section One of the FHSQ with other studies relating to the foot arch is by no means easy, due to differences in the criteria and variations in the protocols concerning the inclusion and exclusion of participants. However, it should be noted that other studies have revealed a link between foot arch height and lower limb injuries, variations in plantar pressure when walking and a variety of social and cultural factors 32 ^-^ 34.

In the sphere of health-related quality of life in general, the scores for Section Two show an impact on overall health, where mean scores were lower than for the other three domains, regardless of the effect of weight, height or BMI. These results indicate, as far as general health is concerned, that when a person, whatever their foot type may be, records a low score they may experience greater limitations in carrying out a wide range of physical activities, become socially isolated and lack the energy to participate in activities. These findings coincide with those of Irving et al. reported in their study of chronic heel pain 25. Neither age, gender nor BMI percentile appear to bear any relation to foot health related quality of life scores and, in line with other studies, no significant differences were found between gender and body weight in the schoolchildren taking part in the study 35 ^,^ 36 **.**

## Conclusions

Comparison of the scores obtained reveal that arch height has a negative impact on quality of life. Since there is a dearth of evidence regarding the aetiology and treatment of foot diseases and deformities, these results highlight the need to implement programmes to promote foot health and to continue research into this commonly occurring disabling condition.
